# Adopting Quality Criteria for Websites Providing Medical Information About Rare Diseases

**DOI:** 10.2196/ijmr.5822

**Published:** 2016-08-25

**Authors:** Frédéric Pauer, Jens Göbel, Holger Storf, Svenja Litzkendorf, Ana Babac, Martin Frank, Verena Lührs, Franziska Schauer, Jörg Schmidtke, Lisa Biehl, Thomas OF Wagner, Frank Ückert, Johann-Matthias Graf von der Schulenburg, Tobias Hartz

**Affiliations:** ^1^ Center for Health Economics Research Hannover (CHERH) Leibniz University Hannover Hannover Germany; ^2^ Medical Informatics Group (MIG) University Hospital Frankfurt Frankfurt am Main Germany; ^3^ Centre for Quality and Management in Healthcare Medical Association of Lower Saxony Hannover Germany; ^4^ Department of Dermatology Medical Center University of Freiburg Freiburg im Breisgau Germany; ^5^ Orphanet Deutschland Center for Rare Diseases Hannover Medical School (MHH) Hannover Germany; ^6^ German Alliance of Chronic Rare Diseases (ACHSE) e.V. Berlin Germany; ^7^ Frankfurt Center for Rare Diseases (FRZSE) University Hospital Frankfurt Frankfurt am Main Germany; ^8^ Department of Medical Informatics for Translational Oncology German Cancer Research Center (DKFZ) Heidelberg Germany

**Keywords:** rare diseases, self-help groups, Internet, health information exchange, quality indicators

## Abstract

**Background:**

The European Union considers diseases to be rare when they affect less than 5 in 10,000 people. It is estimated that there are between 5000 and 8000 different rare diseases. Consistent with this diversity, the quality of information available on the Web varies considerably. Thus, quality criteria for websites about rare diseases are needed.

**Objective:**

The objective of this study was to generate a catalog of quality criteria suitable for rare diseases.

**Methods:**

First, relevant certificates and quality recommendations for health information websites were identified through a comprehensive Web search. Second, all considered quality criteria of each certification program and catalog were examined, extracted into an overview table, and analyzed by thematic content. Finally, an interdisciplinary expert group verified the relevant quality criteria.

**Results:**

We identified 9 quality certificates and criteria catalogs for health information websites with 304 single criteria items. Through this, we aggregated 163 various quality criteria, each assigned to one of the following categories: thematic, technical, service, content, and legal. Finally, a consensus about 13 quality criteria for websites offering medical information on rare diseases was determined. Of these categories, 4 (data protection concept, imprint, creation and updating date, and possibility to contact the website provider) were identified as being the most important for publishing medical information about rare diseases.

**Conclusions:**

The large number of different quality criteria appearing within a relatively small number of criteria catalogs shows that the opinion of what is important in the quality of health information differs. In addition, to define useful quality criteria for websites about rare diseases, which are an essential source of information for many patients, a trade-off is necessary between the high standard of quality criteria for health information websites in general and the limited provision of information about some rare diseases. Finally, transparently presented quality assessments can help people to find reliable information and to assess its quality.

## Introduction

The European Union considers diseases to be rare when they affect no more than 5 in 10,000 people. It is estimated that there are between 5000 and 8000 different rare diseases, affecting nearly 30 million people in the European Union and 4 million people in Germany alone [1,2]. Consistent with this diversity, the quality of information available on the Web varies considerably. People searching the Web often find it very difficult to find the right information and to assess its quality [3,4]. With Orphanet [5], an information platform exists, which holds comprehensive and quality-tested information. However, the target group it addresses is potentially specialists rather than patients [6,7]. In keeping with the European Council’s recommendations, Germany has published a National Action Plan for Rare Diseases in August 2013, which will guide and structure actions in the context of rare diseases within their health and social systems [8]. It includes 52 policy proposals. The national project ZIPSE (German: Zentrales Informationsportal über seltene Erkrankungen; English: Central Information Portal about Rare Diseases), initiated by the Federal Ministry of Health, deals with the realization of the plan’s topics 37 to 39, which cover the subject of a central information portal [9]. Hereby, the health and well-being of people with rare diseases should be improved.

The aim of the ZIPSE project is to conceptualize and implement a central information portal about rare diseases in Germany. A centralized access point for quality-tested information appears to be very helpful for people with a rare disease, their relatives, and medical experts [9]. The portal itself does not contain primary information but refers to existing quality-assured information sources. The aim is the provision of an intelligent user guide to relevant and appropriate sources of information [10]. Web-based information and websites about rare diseases will be linked in the information portal. More precisely, a variety of quality-tested websites about rare diseases will be offered to all users. Furthermore, users will be able to search for disease-specific websites and to filter them by quality criteria. Therefore, a method to distinguish high- and low-quality websites needs to be established [10,11]. A number of quality certificates for websites dealing with medical information already exist. Websites with such a certificate demonstrate quality-tested content [3]. It can be hypothesized that existing quality certificates for websites with health information (eg, Health On the Net Foundation Code of Conduct, HONcode; DISCERN; and Stiftung Gesundheit) are rarely used by websites about rare diseases. It can be assumed that patient organizations often provide well-researched and reliable information about rare diseases, but they have limited resources in terms of time and money to present themselves as professionally as other information providers on the Web to fulfill the requirements of existing quality certificates. Furthermore, the providers’ motivation to present themselves professionally is unknown. The quality control process of certificates such as HONcode can be costly and require significant effort owing to stringent requirements. Verifying websites providing medical information about rare diseases using quality criteria can help increase acceptance and signal trustworthiness to patients, relatives, and medical experts. Most existing quality certificates focused on medical information pursue different goals and contain a wide range of different types of quality criteria. Hence, specific quality criteria for websites about rare diseases are needed. The objective of this study was to generate a catalog of quality criteria suitable for rare diseases. Implementing these quality criteria will improve the evaluation and assessment of information about rare diseases for patients, health professionals, and other users of the information portal.

## Methods

**Figure 1:**
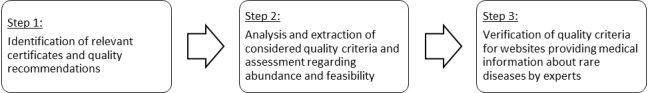
The three steps of the analyzing procedure.

The method we adopted can be regarded as a process divided into 3 steps, as shown in the flowchart in Figure 1.

In step 1, a comprehensive Web search was performed to identify quality certificates and criteria catalogs for websites containing medical or health information. Although we focused on programs and catalogs active in Germany because of its implementation of the information portal about rare diseases, we considered several international sources as well. Quality certificates and criteria catalogs were only included if the quality criteria were published transparently. Furthermore, to be included the certificates and catalogs had to focus on Web-based resources containing medical or health information. Certificates, catalogs, and recommendations were therefore excluded if, for example, they focused only on printed medical information. Additionally, websites about rare diseases were analyzed to identify their quality criteria and their use of quality certificates. These criteria were added if they were not already identified through the Web search. Finally, all identified references were again checked for suitability.

In step 2, the unique criteria of each certification program and catalog were examined, extracted into an overview table, and analyzed by thematic content. Thematic correlations between the criteria were pooled together with an inductive design into major categories. Experts on rare diseases were consulted on the construction of the major categories. Finally, each criterion was assigned to one of the following major categories: thematic, technical, service, content, and legal. Where feasible, the categories were broken down further into groups of criteria. Additionally, experts on rare diseases provided opinions and general information about the importance of each criterion and critical aspects of quality criteria for information about rare diseases. If a criterion was already present in the map, it was not reentered but marked as being part of another criteria catalog. In order to evaluate the importance of a single criterion, its repeated occurrence among different criteria catalogs was examined. Criteria appearing in several catalogs were considered more important, whereas those that were part of a single catalog alone were considered less important. Thus, a hierarchy of the quality criteria appearing in the identified catalogs was constructed, ordered from the criteria appearing the most number of times to those appearing just once.

In step 3, the most important criteria were selected by the project group as preliminary quality criteria. Next, a workshop was held with various experts on website quality and other publications with medical content, experts on health economics and medical informatics, as well as medical experts in the field of rare diseases. A total of 27 experts participated in the workshop—4 of them were professors and 12 graduate doctors. These experts were invited to participate in the group discussion about quality criteria for websites providing medical information about rare diseases. Participants did not receive incentives to attend the workshop and discussion. The relevance and applicability of each quality criterion were discussed, evaluated, and verified by the expert group. The discussion with medical experts as well as experts on the quality of medical information focused on choosing the criteria that should be mandatory for websites offering medical information on rare diseases. Input from medical experts was equally valuable as input from experts on quality of medical information. At the end of the discussion, the experts were expected to arrive at a consensus on the importance of the different quality criteria. Finally, it was decided which of the quality criteria should be mandatory for these websites to be listed on the information portal about rare diseases. Experts from the following institutions participated in the workshop and group discussion:

- German Action Forum Health Information System (afgis e.V.)
- German Alliance of Chronic Rare Diseases (ACHSE e.V.)
- Agency for Quality in Medicine (ÄZQ)
- Federal Ministry of Health Germany (BMG)
- Charité Universitätsmedizin Berlin
- Center for Health Economics Research Hannover (CHERH)
- German Cochrane Center (DCZ)
- Frankfurt Reference Center for Rare Diseases (FRZSE)
- Institute of Medical Biostatistics, Epidemiology and Informatics (IMBEI), University Medical Center Mainz
- Institute for Quality and Efficiency in Health Care (IQWiG)
- Cancer Information Service Heidelberg (KID)
- Hannover Medical School (MHH)
- National Action League for People with Rare Diseases (NAMSE)
- Orphanet Germany
- Public Health Foundation
- Department of Dermatology, Medical Center University of Freiburg
- University Medical Center Hamburg-Eppendorf (UKE)
- Centre for Quality and Management in Healthcare, Medical Association of Lower Saxony (ZQ)

## Results

### Identification of Relevant Certificates

A total of 9 quality certificates and criteria catalogs for websites containing medical or health information were identified. Of these certificates and catalogs, 2 were used internationally; 7 were verified only for German websites. The most common certificate for medical information websites was identified as HONcode [12]. Three further certificates verifying only German websites were identified: afgis Qualitätslogo [13], Stiftung Gesundheit [14], and Medisuch [15]. Additionally, several German, European, and international criteria catalogs were considered: afgis Checkliste für medizinische Websites [16], DISCERN [17], Gute Praxis Gesundheitsinformation [18], NAMSE Kriterien und Standards [19], and Patientenorientierte Krankheitsbeschreibung nach ACHSE-Kriterien [20]. Lastly, the results of a study identifying the most important quality criteria for medical information websites were analyzed [21]. All identified quality catalogs are described in Table 1. Furthermore, the development of quality criteria is an ongoing process, including more detailed quality assurance whereby recent quality catalogs take into account older catalogs and quality certificates. In summary, the identified quality catalogs, certificates, and recommendations show different thematic focuses on the criteria that are considered important to ensure a high quality of health information. Moreover, Table 1 shows that the process of determining the quality of information differs among the identified providers (self-reporting audits vs publicly available information).

**Table 1:** Quality catalogs and quality certificates.

Name	Description	Characteristics	Number of criteria (n=304)
NAMSE Kriterien und Standards^a^ [19]	A discussion paper about quality criteria for enhancing patient information about rare diseases.	It contains requirements for the categories: creation process, results, implementation, and evaluation.	56
HONcode^b^ [12]	As an international certificate, awarded by the Health On the Net Foundation located in Switzerland and established in 1995, it has held NGO^c^ status at the United Nations since 2002. Since 1996, a free certificate for “trusted” sites with medical information was awarded. Since 2015, certification is provided as a paid service. The organization claims that about 8000 medical websites hold their certificate.	Its principles: Information must be authoritative—stating the qualifications of the author. Complementarity—information must supplement and help to support medical advice, not replace it. Privacy—compliance with confidentiality of personal data entered by a website visitor. Assignment—References to sources of information and dates must be present. Verifiability—treatments, products, and services must be supported by balanced, verifiable, scientific information. Transparency and contact information. Disclosure of funding—sponsorship, sponsors, and financial sources must be named. Advertising policy—separation of advertising and editorial content.	55
afgis-Qualitätslogo^d^ [13]	The afgis Qualitätslogo is based on 10 quality categories for transparently provided information, whereby the verification is based on self-reporting audits.	It is based on 10 quality categories for transparently provided information: criteria for transparent information about providers, purpose and target group, authors and information sources, data release, timeliness, and planned maintenance of the information, possibility to give user-feedback, procedure of internal quality assurance, separation of advertisement and editorial contribution, financing and sponsoring, cooperation and networking, and data protection, data transmission, and use of data.	39
afgis Qualitätskriterien [16]	afgis Checkliste für medizinische Websites^e^ is a guideline for providers that want to regenerate websites with medical information content.	It contains essential Web standards for the following categories: timeliness, data protection, design and navigation, medical information, legal aspects, service aspects, search engine, transparency, and access.	35
Gute Praxis Gesundheitsinformation^f^ [18]	A catalog containing quality criteria for the development of health information with a requirement for evidence-based information.	It focuses on the development of health information with a requirement for evidence-based information, which is comprehensible given the expertise of the target group. Thus, the catalog contains different criteria for various target groups.	30
Stiftung Gesundheit^g^ [14]	Awards a seal of approval after checking more than 100 issues, whereby the verification is based on information that is available on the website.	It awards a seal of approval after checking criteria out of the following categories: legal quality, publishing diligence, usability, and search engine optimization.	30
Patientenorientierte Krankheitsbeschreibung nach ACHSE-Kriterien^h^[20]	Contains quality criteria grouped into 5 categories.	It contains quality criteria of the following categories: creation and formal aspects, medical-scientific data and information, disease management, establishment of contact and information about specialties of health professionals, and additional links and references.	28
DISCERN [17]	A tool to evaluate medical publications with a focus on patient information.	It focuses on the following: reliability of the publication and quality of information on treatment alternatives.	19
Medisuch [15]	Provides a certification process and is operated by the institute for quality and transparency of health information.	As a part of its certification process, information providers have to declare that the information provided on the website is not influenced by industrial offers.	12

^a^ NAMSE Kriterien und Standards: NAMSE (National Action League for People with Rare Diseases) criteria and standards (in English).

^b^ HONcode: Health On the Net Foundation Code of Conduct.

^c^ NGO: nongovernmental organization.

^d^ afgis Qualitätslogo: German Action Forum Health Information System (afgis) quality logo (in English).

^e^ afgis Checkliste für medizinische Websites: afgis checklist for medical websites (in English).

^f^ Gute Praxis Gesundheitsinformation: good practice health information (in English).

^g^ Stiftung Gesundheit: Public Health Foundation (in English).

^h^ Patientenorientierte Krankheitsbeschreibung nach ACHSE-Kriterien: patient-oriented description of disease by the criteria of ACHSE (German Alliance of Chronic Rare Diseases) (in English).

### Analysis and Extraction of Quality Criteria

The number of criteria present in the quality certificates is listed in Table 1. The presented number can be higher (or lower) than the official numbers stated by the providers owing to a more detailed valuation of criteria by the project group. The number of criteria ranged from 12 to 56 in the catalogs analyzed. In total, we identified 304 single criteria items. Through this, we aggregated 163 different quality criteria into 5 major categories: thematic, technical, service, content, and legal. The thematic criteria category containing 90 criteria (90/163, 55.2%) was by far the largest, followed by the service category with 26 criteria (26/163, 16.0%), the technical category with 18 (18/163, 11.0%), the legal category with 15 (15/163, 9.2%), and the content category with 14 (14/163, 8.6%). The degree of detail varied among the different criteria catalogs, and while 66 criteria (66/163, 40.5%) were found in multiple catalogs, no criterion was found in all of the certificate definitions or criteria catalogs. The 2 most frequently occurring criteria appeared in 6 of the analyzed catalogs (6/9, 67%). Three criteria appeared in 5 (5/9, 56%) and 13 criteria in 4 of the catalogs (4/9, 44%), whereas 20 criteria appeared in 3 (3/9, 33%) and 28 criteria in 2 of the catalogs (2/9, 22%). The majority of 87 criteria were unique to a single catalog. With the exception of one catalog (Gute Praxis Gesundheitsinformation), each contains a criterion unique to itself. All identified quality criteria are presented in Multimedia Appendix 1. In summary, the number of criteria present in quality certificates and quality catalogs differs. Nevertheless, most catalogs contain a unique criterion not shown elsewhere. The number of quality criteria in each of the major categories varies widely.

### Expert Verification

To assess the relevance of a quality criterion specific to websites offering medical information on rare diseases, different principles were applied. First, criteria appearing in many of the reviewed catalogs were considered more important to ensure a certain level of information quality. This resulted in initially selecting the two most abundant criteria (authors are mentioned and creation and updating dates of information are mentioned) as mandatory for websites to be listed in the information portal ZIPSE. Criteria appearing less often were only selected in consideration with their relevance and their applicability to rare diseases and the targeted websites. This relevance was assessed by checking several properties. If a criterion is applicable, it is to a certain extent defined by its feasibility. Criteria seemingly important to the quality of general medical information may only be adapted to a limited extent. Finally, in the discussion workshop with 27 experts, quality criteria for websites offering medical information on rare diseases were defined. A consensus about the following 13 quality criteria for websites offering medical information on rare diseases was determined:

- Authoring information
- Mentioning of authors
- Mentioning of sources
- Mentioning of creation and update date
- Data security
- Declaration of evidence
- Marking of conflicts of interests
- Consideration of target group
- Evaluation of content
- Review of information
- Characteristics of the website (accessibility)
- Imprint
- Contact opportunity

A decision was made on the quality criteria that should be a mandatory requirement for websites about rare diseases for them to be listed in the information portal. As a legal requirement for all websites, an adequate *data protection concept* as well as an *imprint* is mandatory. Moreover, we identified the *creation and updating date* and the possibility to *contact the website provider* as very important categories for patients with a rare disease.

## Discussion

### Principal Findings

The literature review of quality catalogs, certificates, and recommendations for websites containing medical or health information showed different thematic focuses on criteria that are important for the quality of health information. Interestingly, the investigated certificates reveal a great variety of quality criteria used by the common certificates. There is also a wide range of quality criteria where the degree of detail varied among the different criteria catalogs. Furthermore, the process for determining the quality of websites differs among the identified providers (self-reporting audits, eg, [13] vs publicly available information, eg, [14]). The classification of the quality criteria into the major categories, thematic, technical, service, content, and legal, showed that the number of quality criteria in each category varies widely. The presence of a larger number of quality criteria in one category does not necessarily indicate a greater relevance of the category. It is rather an indication that this category can be investigated more thoroughly than categories with a smaller number of different criteria [12].

Defined quality criteria for websites about rare diseases were coordinated and verified by a multidisciplinary expert group to ensure the quality of the information provided. These quality criteria will be applied for registration of websites on the portal about rare diseases. Out of the 13 verified quality criteria for websites about rare diseases, 4 were identified to be mandatory for registration to the information portal. First, as a legal requirement for all websites an adequate *data protection concept* and an *imprint* are mandatory. Moreover, *creation and updating date* and *possibility to contact the website provider* were identified as very important categories for patients with a rare disease. The documentation of the creation and updating date of information is especially important owing to rapid advances in the development of information and to demonstrate the latest research findings [22]. The possibility to contact the website provider is also an important quality aspect for these websites. Particularly, if there is limited information elsewhere, patients, health professionals, and other users can offer the provider advice or suggestions for improvement or ask for more precise information about a rare disease [23]. These 4 categories are mandatory for registration to the information portal and for linking to medical information about rare diseases. Fulfillment of the remaining 9 categories is optional. Nonetheless, these categories are still important for quality-tested information about rare diseases. To achieve transparency, it would be beneficial to publish the degree to which the websites fulfill these categories. In particular, information on the characteristics of the website, such as its accessibility, is important for many patients [24]. Thus, the fulfillment of each single low-barrier criterion needs to be shown transparently.

Using quality criteria to verify websites providing medical information about rare diseases can help to improve their acceptance and signal trustworthiness to patients, relatives, and medical experts [3]. In further studies, all selected quality criteria will be transferred to a so-called self-disclosure questionnaire. These questions will then be used to assess the quality of rare disease websites. The results from the first evaluation of these can help to improve and adjust the quality assessment process of the information portal. Moreover, we can evaluate and test the assumptions made at the beginning:

- Do patient organizations provide well-researched and reliable information about rare diseases?
- Do they present themselves as professionally as other information providers on the Web to fulfill the requirements of existing quality certificates?
- Do websites with little content and a small editorial staff hold high-quality information?

A further problem for investigation is the availability of robust evidence of information on rare diseases. Providing evidence for the source of information is a requirement often sought to ensure a piece of information is well researched. However, with merely 5 in 10,000 people affected by rare diseases, it is almost impossible to collect sufficient data to statistically test a hypothesis. It could be argued that a single proven case is also a form of evidence, albeit a very thin one. However, as long as no other data exist, it is still the best evidence available [25]. There are also important implications for future research from analysis of those categories where we identified a lower number of different criteria. New detailed quality criteria on these categories may help improve the discussion on quality of websites providing medical information.

### Limitations

Despite our focus on programs and catalogs active in Germany, we identified a large number and variety of different quality criteria. As with other quality catalogs, the defined criteria cannot verify the thematic content of health information. These criteria simply verify factors influencing the thematic content, as well as the quality of the website itself. A more complex and expensive solution to verify the heterogeneous information about rare diseases would be for medical experts to verify and highlight single articles of listed websites about rare diseases in the information portal. The defined quality criteria for such websites were verified by the participants of a workshop. Although this workshop was held with 27 renowned and excellent experts on website quality and other publications with medical content, experts on health economics and medical informatics, as well as medical experts in the field of rare diseases, subjectivity in their decision-making process cannot be ruled out.

### Conclusions

The relatively low intersection of criteria appearing in the different criteria catalogs shows that the opinion of what is important concerning quality of medical information differs. For the development of useful quality criteria for websites about rare diseases, a trade-off between the high standard of quality criteria for general health information and the provision of limited existing information about rare diseases, which is essential for many patients, appears unavoidable. Providing defined quality criteria for websites about rare diseases can help seekers to find reliable information and to assess its quality [3,4]. Accepted criteria for websites with information about rare diseases, which allow for a minimum of quality control while keeping the workload reasonable, have been defined. In summary, 13 categories with quality criteria were defined by a group consisting of medical experts as well as experts on the quality of medical information. Fulfillment of 4 of these categories (*data protection concept*, *imprint*, *creation and updating date*, and *possibility to contact the website provider*) was identified as being mandatory for registration to the information portal and for publishing medical information about rare diseases. With the help of these quality criteria, we can evaluate, for instance, the quality of information provided by rare disease self-help groups or other information providers.

## Appendix

Multimedia Appendix 1 Quality criteria for health information websites.

## Abbreviations

ACHSE: German Alliance of Chronic Rare Diseases
afgis: German Action Forum Health Information System
BMG: Federal Ministry of Health Germany
e.V: registered society
HONcode: Health On the Net Foundation Code of Conduct
NAMSE: National Action League for People with Rare Diseases
ZIPSE: Central Information Portal about Rare Diseases

## References

[1] Commission of the European Communities (2008). Europa.

[2] Federal Ministry of Health Germany Bundesministerium für Gesundheit.

[3] Breckons M, Jones R, Morris J, Richardson J (2008). What do evaluation instruments tell us about the quality of complementary medicine information on the internet?. J Med Internet Res.

[4] Dubowicz A, Schulz PJ (2015). Medical information on the internet: a tool for measuring consumer perception of quality aspects. Interact J Med Res.

[5] Orphanet Orphadata.

[6] Maiella S, Rath A, Angin C, Mousson F, Kremp O (2013). [Orphanet and its consortium: where to find expert-validated information on rare diseases]. Rev Neurol (Paris).

[7] (2013). Orpha.

[8] Aymé S (2012). State of the art of rare disease activities in Europe: a EUCERD perspective. Orphanet J Rare Dis.

[9] NAMSE (2013). National action league for people with rare diseases.

[10] ZIPSE.

[11] Fahy E, Hardikar R, Fox A, Mackay S (2014). Quality of patient health information on the Internet: reviewing a complex and evolving landscape. Australas Med J.

[12] HON Health On the Net Foundation.

[13] Afgis e.V. (2016). Afgis.

[14] Stiftung Gesundheit stiftung-gesundheit.

[15] Medisuch Medisuch.

[16] Afgis e. V. (2010). Afgis e. V. Checkliste für medizinische Websites.

[17] DISCERN discern.

[18] Deutsches Netzwerk evidenzbasierte Medizin ebm-netzwerk.

[19] NAMSE NAMSE.

[20] (2016). ACHSE e.V.

[21] Eysenbach G, Powell J, Kuss O, Sa ER (2002). Empirical studies assessing the quality of health information for consumers on the world wide web: a systematic review. JAMA.

[22] Neumark Y, Flum L, Lopez-Quintero C, Shtarkshall R (2012). Quality of online health information about oral contraceptives from Hebrew-language websites. Isr J Health Policy Res.

[23] Baas M, Huisman S, van HJ, Koekkoek G, Laan H, Hennekam RC (2015). Building treasures for rare disorders. Eur J Med Genet.

[24] Barbara AM, Dobbins M, Haynes RB, Iorio A, Lavis JN, Raina P, Levinson AJ (2016). The McMaster Optimal Aging Portal: Usability Evaluation of a Unique Evidence-Based Health Information Website. JMIR Hum Factors.

[25] Casali PG, Bruzzi P, Bogaerts J, Blay J, Rare Cancers Europe (RCE) Consensus Panel (2015). Rare Cancers Europe (RCE) methodological recommendations for clinical studies in rare cancers: a European consensus position paper. Ann Oncol.

