# Supplementary material for: Adopting Quality Criteria for Websites Providing Medical Information About Rare Diseases
Source: Interact J Med Res. 2016 Aug 25;5(3):e24. doi: 10.2196/ijmr.5822 (PMC5016623; doi:10.2196/ijmr.5822)
Supplement: Multimedia Appendix 1 [file ijmr_v5i3e24_app1.pdf]

Appendix A

Quality criteria for health information websites (quality criteria contained in catalogue number x)

	1	2	3	4	5	6	7	8	9	
**Technical criteria—General**										
Website is understandable with the help of current technology (1) (3) (5)	1		1		1					
Structure and layout are target group-specific (3)			1							
No need for particular plugins or other components (3) (5)			1		1					
No need for a broadband connection (3) (5)			1		1					
No need for a particular screen resolution (3) (5)			1		1					
No impairment for reading due to color design and contrast (3) (5)			1		1					
Uniform appearance of recurrent elements (5)					1					
Website is printable and readable (5)					1					
Full-text search is available (5)					1					
Character size is adaptable (via Browser or site function) (5)					1					
No use of frames (5)					1					
Layout is traceable for search engines (5)					1					
**Technical criteria—Navigation**										
Navigation is operational (5)					1					
Navigation is consistent (5)					1					
Menu items do not contain more than 7 sub items (5)					1					
**Technical criteria—Links**										
Links are operational (5)					1					
Links are clearly visible (5)					1					
Links are at each home page (5)					1					
**Total—Technical criteria**	**1**	**0**	**6**	**0**	**17**	**0**	**0**	**0**	**0**	**24**
**Legal criteria—General**										
Imprint created in pursuance with § 5 TMG (2) (3) (5)		1	1		1					
Compliance with the competition law (3)			1							
Compliance with press law (3)			1							
**Legal criteria—Advertising**										
Compliance with pharmaceutical-advertising law (3)			1							
Information about use of advertising via "Web-Policy" (1)	1									
Advertising is clearly marked as such (1) (2) (3) (4)	1	1	1	1						
Unpaid advertising is marked as such and is separated from other content (1) (2) (3) (4)	1	1	1	1						
If there is no advertising, there will be a declaration that the website is not financed by advertising (1)	1									

If there is advertising from third-party providers (e. g. Google), there will be a declaration that this is not an influence on the advertising by the website operator (1)	1									
If there is user created content, it will be clarified if users are allowed to advertise or not (1)	1									
**Legal criteria—Data security**										
By means of a privacy policy there is information about handling of personal data (1) (2) (3) (5)	1	1	1		1					
Information about potentially unprotected E-Mail communication is provided (2) (5)		1			1					
Information about the use of analysis tools (GA, Piwik) (1) (2) (5)	1	1			1					
Confidential handling of data (1) (2)	1	1								
Compliance with medical data security of other countries (list of these countries required)										
**Total—Legal criteria**	**9**	**7**	**7**	**2**	**4**	**0**	**0**	**0**	**0**	**29**
**Content**										
User created content is recognizable (3)			1							
Information is provided if content is moderated by somebody (1)	1									
Frequency of visits of moderators is displayed (1)	1									
Information is provided if all prior users are listed as medical professionals (1)	1									
Content by medical professionals is marked as such (1)	1									
Respect and truthfulness (1)	1									
Information about infringement proceedings by users (1)	1									
Inaccurate content will be deleted as soon as possible (3)			1							
User created content is tagged with a creation date (1)	1									
Minimum age for subscribers is listed and will be fulfilled (1)	1									
**Content—Notes**										
Note that all contributions are visible for all visitors (1)	1									
Information about the possibility to delete or change contributions at a later date (1)	1									
Note that every user must include references unless it is a personal experience (1)	1									
Note that users should only publish content if they know that it is truth										
**Total—Content criteria**	**11**	**0**	**2**	**0**	**0**	**0**	**0**	**0**	**0**	**13**
**Service criteria**										
Users have the opportunity to give feedback or to contact the website operator (1) (2) (3)	1	1	1							
Comprehensive evaluation of the information by creation date (8)								1		
A contact form or E-Mail address is easy to find(1)	1									
Information about processing time of requests (2) (5)		1			1					
Requests were answered individually and in a short time (1)	1									
Registered users have special possibilities to contact the website operator (user created content) (1)	1									
The standards of user created content are easily traceable (1)	1									

Links to other (external) information (2) (5) (9)		1			1				1	
Information about additional literature (6) (8) (9)						1		1	1	
Information about additional options for counseling and contact (6) (7) (8) (9)						1	1	1	1	
Information about structure of medical supply (6) (8) (9)						1		1	1	
Website is traceable by search engines (Bing, Google) (2) (5)		1			1					
Website is traceable by special search engines (MedHunt, medinfo) (2) (5)		1			1					
Content is in the local language (8) (9)								1	1	
Translation in other languages is encouraged (8)								1		
Information is available on the website as well as in print (8)								1		
The description of diseases is easy to understand and free of charge (9)									1	
**Procedures for quality assurance are documented**										
Procedure for designation of persons involved (8)								1		
Type of involvement (8)								1		
Editorial process (8)								1		
Search (search strategies and results) (8)								1		
Type of inclusion of patient experiences (8)								1		
Explanation of the selection procedure of specialized services (8)								1		
Explanation of the selection procedure of references (2)		1								
Explanation of the kind of updating (2)		1								
Explanation of the type of identification of authors (2)		1								
**Total—Service criteria**	**5**	**8**	**1**	**0**	**4**	**3**	**1**	**13**	**6**	**41**
**Thematic criteria—Topicality**										
Creation and updating date of information is mentioned (1) (2) (5) (6) (8) (9)	1	1			1	1		1	1	
**Transparency**										
Owner of the website is mentioned (1) (2) (3)	1	1	1							
Objectives of the owner are mentioned (1) (2)	1	1								
Sponsors and other supporters are mentioned (1) (2) (3) (5)	1	1	1		1					
Information about cooperation and networks (2) (3)		1	1							
**Clear separation between content and advertising**										
Disclosure of data capture of users (1) (2) (3)	1	1	1							
Disclosure of the use of cookies (1) (3)	1		1							
Financing and donors are mentioned (1) (2) (7) (8)	1	1					1	1		
Conflicts of interests are mentioned (1) (2) (7) (8)	1	1					1	1		
No operational or editorial participation of pharmaceutical, medical technology or food industry (4)				1						
Editorial guidelines are mentioned (2)		1								
If there is user created content, it will be mentioned if there is a voluntarily working moderator (1)	1									
Donations from the pharmaceutical, medical technology or food industry are below a certain threshold (4)				1						
No direct or indirect influence over the content by the industry (4)				1						

**Content—Target group**									
Intended target group becomes clear (1) (2) (6)	1	1				1			
Information is adjusted for patients (2) (4) (5) (8) (9)		1		1	1			1	1
Information is adjusted for the target group (3) (6) (7) (8)			1			1	1	1	
Information is understandable for non-professionals (2)		1							
**Content—Targets**									
Targets become clear (1) (2) (3) (6)	1	1	1			1			
Targets are achieved (6)						1			
Mission statement is explained (1)	1								
**Content—Authors**									
Authors are mentioned (1) (2) (3) (7) (8) (9)	1	1	1				1	1	1
Patients (or patient representatives) and medical professionals are part of the authorship (7) (8) (9)							1	1	1
Type of involvement is mentioned (7) (9)							1		1
**General information about authors are mentioned**									
Qualification of the authors are mentioned (1) (2) (3)	1	1	1						
Qualification of the persons responsible for the content are mentioned (1) (2)	1	1							
Medical specialties of medical professionals are mentioned (1)	1								
Non-professional authors are clearly mentioned (1)	1								
Potential conflicts of interest of the authors are mentioned (2) (7) (8)		1					1	1	
**Sources**									
All used sources are mentioned (ideally as a list of references) (1) (2) (5) (6) (8)	1	1			1	1		1	
Sources are linked (if possible) (1)	1								
Personal experiences are mentioned as a source (1) (2)	1	1							
Creation date of the most important sources is mentioned (6)						1			
**Formal**									
Foreign words are explained (2) (5) (8) (9)		1			1			1	1
Abbreviations are explained at the first time of occurrence (2) (5)		1			1				
Abbreviations of professional title and academic grades are explained (1)	1								
Brand names are marked as such (e. g. with ®) (1)	1								
**Online consultation**									
No remote diagnosis/tele diagnosis (1)	1								
Online consultation is not (much) more expansive than consultation of a health professional (1)	1								
Online consultation is just for people living in the same country as the website provider (1)	1								
**Content—Information**									
Emphasis on information about patient-relevant health information (4) (7)				1			1		
Information about marketing is subordinated (4)				1					
No adaption of writings from companies without revision (4)				1					
Information is action and practically orientated (5)					1				

Information is objectively correct (2) (3) (5) (9)		1	1		1				1
Claims are proved (1)	1								
Medical information is evidenced based (1) (2) (5) (7) (8)	1	1			1		1	1	
Need for evidence is mentioned (7) (8)							1	1	
Information is clearly structured (2) (3) (5) (9)		1	1		1				1
Information is systematically researched, edited and evaluated (7) (8)							1	1	
The content is balanced and without any influence (Exception: Websites about product information) (3) (6) (7) (8)			1			1	1	1	
Content is comprehensive (9)									1
Content is understandable (4) (7) (9)				1			1		1
Content is formulated neutrally (7) (8)							1	1	
Products and brands are not mentioned (8)								1	
Risks are comprehensibly described (7) (8)							1	1	
Information from studies which has not yet been proven is mentioned where appropriate (6) (7) (8)						1	1	1	
Natural course of a disease is described (8)								1	
The content is influenced by experiences with medical professionals (about medical care, course of diseases and disease management) (8)								1	
Information about disease management as well as psychological and pedagogical support by patients and relatives (and if possible by studies) is given (8)								1	
Description of diagnostic test including positive and negative prediction values (8)								1	
Basis of shared decision-making (6)						1			
Basis of counseling sessions (8)								1	
Addresses and evaluation have an appropriate extent (4)				1					
Selection criteria for addresses are mentioned (4)				1					
Public consultation phase before finalization of information (8)								1	
**Notes**									
Note that information on websites are not substitute medical findings by an medical professional (1)	1								
**Topics**									
Knowledge and limitation of knowledge (7) (8)							1	1	
Frequency (8) (9)								1	1
Causes (7) (8) (9)							1	1	1
Symptoms (7) (8) (9)							1	1	1
Diagnosis (7) (8)							1	1	
Differential diagnosis (9)									1
Progression of the disease (7) (8) (9)							1	1	1
Disease management (7) (9)							1		1
Life planning (9)									1
Forecast/prognosis (9)									1
Screening/early recognition (7) (8) (9)							1	1	1
Prevention (7) (8) (9)							1	1	1

Genetic counseling (8) (9)								1	1	
Genetic diagnosis (8)								1		
Testing methods processing diagnosis (8) (9)								1	1	
Treatment results (7) (8)							1	1		
**Therapy**										
Mode of action of treatment processes are described (6) (7) (8)						1	1	1		
Utility of treatment processes are described (6) (7) (8)						1	1	1		
Risks of treatment processes are described (3) (6) (7) (8)			1			1	1	1		
Consequences of non-treatment are described (6) (7) (8)						1	1	1		
Influence of treatment processes to life quality are described (6) (8)						1		1		
Existence of further treatment processes is mentioned (Exception: Websites about product information) (1) (3) (6) (8)	1		1			1		1		
National differences in treatment options are mentioned (8)								1		
**Total—Content criteria**	**29**	**24**	**14**	**10**	**10**	**16**	**29**	**43**	**22**	**197**
**Total**	**55**	**39**	**30**	**12**	**35**	**19**	**30**	**56**	**28**	**304**
